# Cell Subsampling Recovers Probative DNA Profile Information from Unresolvable/Undetectable Minor Donors in Mixtures

**DOI:** 10.3390/genes13071117

**Published:** 2022-06-22

**Authors:** Kaitlin Huffman, Erin Hanson, Jack Ballantyne

**Affiliations:** 1 Graduate Program in Chemistry, Department of Chemistry, University of Central Florida, P.O. Box 162366, Orlando, FL 32816-2366, USA; huffmank13@knights.ucf.edu (K.H.); erin.hanson@ucf.edu (E.H.); 2 National Center for Forensic Science, P.O. Box 162367, Orlando, FL 32816-2367, USA

**Keywords:** unresolvable minor donor, mini-mixtures, few cell PG analysis

## Abstract

When a minor DNA component to a binary mixture is present at a weight ratio of approximately 1:50 or less, the presence of this minor donor is undetectable (or barely detectable) by standard mixture deconvolution approaches. In an attempt to retrieve probative minor donor DNA profile information, multiple quintuple cell subsamples were collected from a 1:50 DNA mixture using direct single cell subsampling (DSCS) paired with probabilistic genotyping (PG), the latter validated for use with single or few cells. DSCS employs a simplified micromanipulation technique paired with an enhanced DNA profiling approach, involving direct cell lysis and a sensitive PCR process, to genotype individual cells. Multiple five-cell subsamples were used to interrogate sufficient cells from the mixture such that some of the created 5-cell “mini-mixture” subsamples contained a cell from the minor donor. The latter mini-mixture subsamples, which now comprised weight ratios of 1:4 as opposed to the bulk mixture 1:50, were analyzed with the PG systems STRmix^TM^ and EuroForMix resulting in a significant probative gain of information, (LR ≅ 10^11^, compared to standard bulk mixture PG methods, LR ≅ 10^1^–10^2^).

## 1. Introduction

Standard DNA mixture analysis typically targets 0.5 ng to 1 ng DNA for amplification from a bulk DNA extraction process that basically “homogenizes” the mixture components. This can be problematic in instances in which one donor’s DNA is present in such a large proportional concentration that it effectively masks the presence of a minor donor due to PCR reaction kinetics. In many cases, alternative approaches such as physical separation by way of differential extraction [1] or conducting Y-STR analysis, if the major donor is female and the minor donor is male, can result in the recovery of probative DNA profile information [2,3]. However, the differential extraction process is not conducive to all forensic sample types such as non-semen containing samples, and additionally, if both donors are male or if the minor donor to a mixture is female, then Y-STR analysis is not likely to be of benefit.

Probabilistic genotyping (PG), until recently, has primarily been applied to bulk samples extracted from DNA stains, and although highly useful, does not always result in probative DNA information for all donors to a mixture due to the presence of allele overlap, artifacts such as stutter, and in the case of this paper, the extreme minor presence of one of the donors [4,5,6,7,8]. Low-level minor DNA contributors to a mixture that are not (or are barely) detectable by standard methods could thus benefit from a sample subsampling method to retrieve the otherwise totally (or almost totally) lost DNA profile information.

A previous report has shown that isolation of multiple groups of cells from mixtures results in sub-populations with differing constituent weight ratios [6] that, in combination, can recover more DNA profile information, while others have demonstrated the potential to obtain highly probative single source samples from mixtures following analysis of recovered single or multiple cell subsets [7,8,9,10,11,12,13,14,15]. One of these latter physical cell recovery methods (direct single cell subsampling, DSCS) allows for multiple physical subsamplings of individual cells (or cell subsets) from complex cellular DNA mixtures using a stereomicroscope and tungsten needle. The application of enhanced direct PCR followed by appropriately validated probabilistic genotyping approaches to these single or few cell mixture subsamples has resulted in highly probative likelihood ratio (LR) values for true donors to complex equimolar mixtures [8].

The purpose of this study was to develop a cell subsampling strategy to enable the detection and analysis of a minor donor to a binary mixture that would otherwise be un-detectable or barely detectable using standard mixture interpretation methods. In the present work, the direct single cell subsampling approach, which utilizes the simplified micromanipulation technique paired with direct cell lysis and a sensitive PCR process to genotype individual cells, was used to collect five-cell subsamples from a complex binary mixture (1:50) in which the minor donor was virtually undetectable by standard methods. This resulted in new mini-mixture subsamples (with weight ratios of 1:4 as opposed to 1:50) that could then be analyzed and interpreted by way of probabilistic genotyping, resulting in a gain of significant probative DNA information from the minor donor.

## 2. Methods

### 2.1. Sample Collection

Buccal cell samples were collected from volunteers using procedures approved by the University of Central Florida’s Institutional Review Board. Samples were obtained from each volunteer by swabbing the inside of the mouth and cheek with a sterile cotton swab.

### 2.2. Slide Creation

#### 2.2.1. Mixture Slide Creation

Freshly collected or dried/frozen buccal swabs from individual donors (“SA79” and “S3”) were agitated in 300 μL of TE^−4^, and the resulting solutions were centrifuged at 300 RCF for 7 min to create an epithelial cell pellet. The supernatants were discarded, and 300 μL of TE^−4^ was added to each cell pellet to create a cell suspension. The Countess™ II FL (ThermoFisher Scientific, Carlsbad, CA, USA) automated cell counter was used according to manufacturer recommended protocols to determine the cell concentration of each cell suspension. The appropriate volumes of each donor cell suspension were mixed to achieve the desired donor mixture ratio (i.e., 1:50). The mixture cell suspension was stored frozen (4 °C) before use.

Gel-Pak^®^ Gel-Film^®^ (WF, x8 retention level, Hayward, CA, USA) was affixed to a clean glass microscope slide via its adhesive backing, and the clear protective covering removed. Once the desired mixture was created, 60 μL of the mixture solution was pipetted onto the Gel-Film^®^ microscope slide and spread out using a sterile swab. The slide was then stained with Trypan Blue for 1–2 min and gently rinsed with nuclease-free water. Slides were air-dried overnight.

#### 2.2.2. 3M^TM^ Adhesive Slide Creation

A 3M™ (Allied Electronics, Fort Worth, Texas, USA) adhesive slide reservoir was prepared prior to cell collection, in which double-sided tape was used to attach the 3M™ adhesive to a clean glass microscope slide. The 3M™ adhesive backing was removed, and the slide was stored in a desiccator until needed [16,17].

### 2.3. Cell Recovery

Cells were visualized using a Leica M205C stereomicroscope (190–240× magnification) and collected directly into 5 μL PunchSolution^TM^ (Promega, Madison, WI, USA) present in sterile 0.2 mL PCR flat-cap tubes by way of a sterile tungsten needle and water-soluble 3M™ adhesive. The tungsten needle was used to acquire a small ball of 3M™ adhesive, which was then utilized to adhere selected visualized cells from the mixture slide. Still under the microscope, the adhesive-tipped tungsten needle containing adhered cells was inserted into an amplification tube (0.2 mL) containing PunchSolution^TM^ (Promega, Madison, WI, USA) until the 3M™ adhesive is observed to solubilize [16,17]. An open access, on-line detailed video demonstration of the cell capture and recovery method can be found in reference [16].

### 2.4. Direct Lysis/Autosomal Short Tandem Repeat (STR) Amplification of Cells

Cells were collected directly into 5 μL PunchSolution^TM^ (Promega, Madison, WI, USA) in 0.2 mL PCR flat-cap tubes. Samples were incubated until the PunchSolution^TM^ (Promega, Madison, WI, USA) evaporated using a lysis protocol of 70 °C → 30 min. After lysis, GlobalFiler^TM^ Express reaction mix was added to each mixture consisting of 2 μL PCR mix, 2 μL primer mix, 1 μL 5X AmpSolution^TM^ (Promega, Madison, WI, USA). Samples were amplified using a modified protocol of 95 °C → 1 min; 32 cycles: 94 °C → 3 sec, 60 °C → 30 sec; 60 °C → 8 min; 4 °C → hold.

### 2.5. Donor Reference Samples and Bulk Mixtures

#### 2.5.1. DNA Isolation and Quantitation

DNA was extracted from reference buccal swabs and 60 μL of the mixture cell suspension using the AutoMate *Express*™ Forensic DNA Extraction System (ThermoFisher Scientific, Carlsbad, CA, USA) using the PrepFiler Express™ Forensic DNA Extraction Kits (ThermoFisher Scientific, Carlsbad, CA, USA). An extraction blank was included with each extraction set performed.

#### 2.5.2. Autosomal STR Amplification (Reference Samples and Bulk Mixture)

Amplification of the donor reference samples and the bulk DNA mixtures was conducted using the GlobalFiler^TM^ (ThermoFisher Scientific, Carlsbad, CA, USA) amplification kit according to the manufacturer’s recommended protocols. One nanogram of input DNA was used with an amplification protocol of 95 °C → 1 min; 29 cycles: 94 °C → 10 sec, 59 °C → 90 sec; 60 °C → 10 min; 4 °C → hold. Positive and negative amplification controls were included in each amplification.

### 2.6. PCR Product Detection

One microliter of GlobalFiler™ or GlobalFiler™ Express (ThermoFisher Scientific, Carlsbad, CA, USA) amplified product was added to a mixture comprised of 9.5 μL Hi-Di™ formamide (ThermoFisher Scientific, Carlsbad, CA, USA) and 0.5 μL GeneScan™ 600 LIZ^®^ size standard (ThermoFisher Scientific, Carlsbad, CA, USA). Samples were injected on an Applied Biosystems’ 3500 Genetic Analyzer using POP-4™ polymer and Module J6 (15 s injection, 1.2 kV, 60 °C) and analyzed using GeneMapper™ ID-X v1.6 software (ThermoFisher Scientific, Carlsbad, CA, USA).

### 2.7. Probabilistic Genotyping (PG)

#### 2.7.1. Standard Bulk Mixture Probabilistic Genotyping

Probabilistic Genotyping Software STRmix™ v2.8 and EuroForMix v3.1.0 (Quantitative LR MLE based) was previously internally validated for use with standard bulk mixtures and reference samples. Each sample was analyzed according to the known number of donors. With STRmix^TM^, the sub-source log (LR) was reported. The FBI Caucasian database was used for all allele frequencies in all mixture experiments [8].

#### 2.7.2. DSCS Probabilistic Genotyping

Probabilistic Genotyping Software STRmix™ v2.8 [8] and EuroForMix v3.1.0 (Quantitative LR MLE based) was previously internally validated for use with 1–5 cell subsamples. Each subsample was either run as single source (i.e., $LR=\frac{P_{r}\left(E|\;POI\right)}{P_{r}\left(\left.E\right|\;unknown\;individual\right)}$) or, when appropriate, as a 2-person mixture (i.e., $LR=\frac{P_{r}\left(E|\;unknown\;individual+POI\right)}{P_{r}\left(\left.E\right|\;2\;unknown\;individuals\right)}$). When EuroForMix was utilized, all sub-samples were modeled with degradation, forward, and reverse stutter unless the model failed, in which case, the sample would be modeled without degradation. Subsamples that returned inclusionary LRs (i.e., log(LR) ≥ 1) for the minor donor were utilized for replicate analysis.

### 2.8. Description of DSCS Method

**Figure 1:**
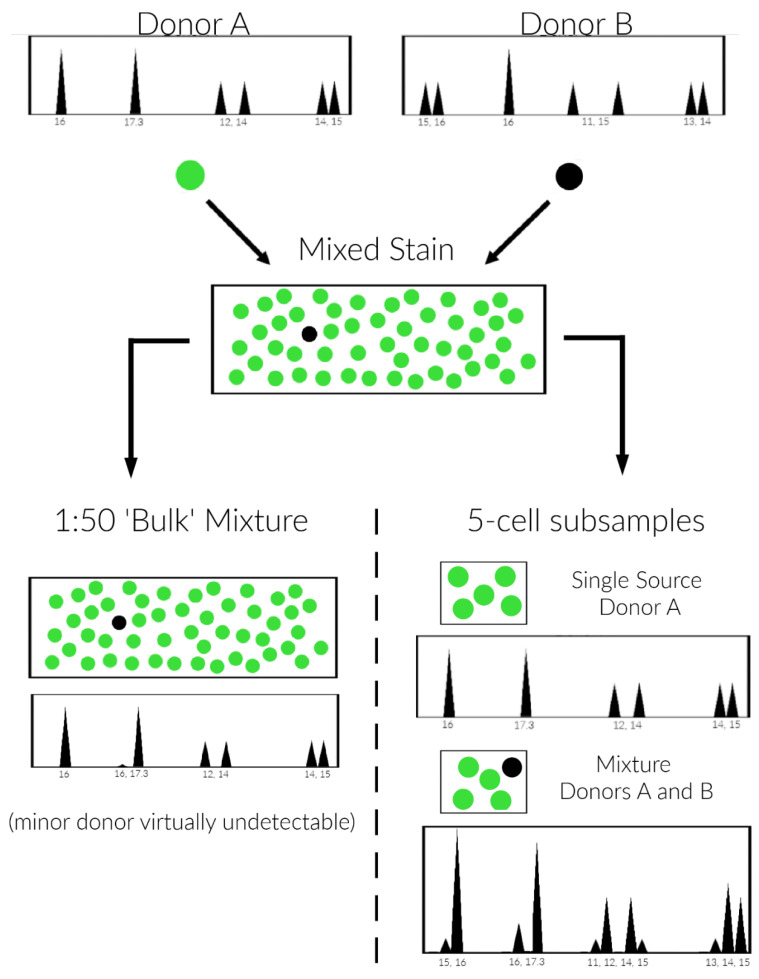
Undetectable/unresolvable minor donor analysis scheme. Standard “bulk” sampling results in a DNA profile that appears likely single source for individual A where individual B’s genotype cannot be distinguished (left side). The minor donor (B) is undetectable/barely detectable (left side). Simplified micromanipulation subsampling (DSCS) of the same mixture using a 5-cell subsampling size results in single source profiles of individual A and 1:4 mini-mixture samples of individuals A and B (right side, A is major donor and B is the minor donor)). These mini-mixture subsamples are then analyzed with PG validated for one or few cells.

An infographic of the quintuple cell DSCS method is provided in Figure 1, which considers a binary 1:50 mixture where standard “bulk” sampling results in a DNA profile that appears likely single source for individual A. The minor donor (B) is undetectable/barely detectable (left side). DSCS subsampling of the same mixture using a 5-cell subsampling size results in single source profiles of individual A and 1:4 mini-mixture samples of individuals A and B (right side, A is major donor and B is the minor donor). These mini-mixture subsamples are then analyzed with PG validated for one or few cells.

## 3. Results and Discussion

### 3.1. Subsampling Strategy to Recover Minor Donor DNA Profiles

To determine at what mixture ratio a minor donor would become undetectable using our standard STR analysis system, two-person DNA mixtures ranging from tenfold ratios of 1:20 to 1:50 were prepared and analyzed. It was determined that a donor ratio of approximately 1:50 was the point at which a minor donor became virtually undetectable using standard methods. Thus, we proceeded to develop and investigate a DSCS strategy that would be applicable to undetectable/unresolvable mixtures with a minor donor ratio of approximately 1/50 but which would also probably be applicable to mixtures with lower weight ratios of down to approximately 1/100. The objective of the subsampling is primarily to interrogate a sufficiently large number of mixture cells such that some from the minor donor are recovered and able to be genotyped. The simplest approach would be to remove single cells as per the standard DSCS approach [6,7], which preferably requires replicates (usually at least 3) for optimal LR recovery. Therefore with ~1: 50 minor donor (~2%), at least 150 single cell subsamples would have to be recovered and genotyped (and for a 1:100 mixture, 300 subsamples), but the reagent cost would be prohibitive. An alternative is to interrogate a larger number of cells by increasing the number of cells in a subsample from one to five and increasing the number of subsamplings themselves. Therefore, collections of five-cell subsamples were chosen to interrogate a larger number of cells (5×) compared single cells, thereby increasing the probability of obtaining subsamples with cells from the minor donor. These five-cell subsamples would mainly comprise subsamples that were single source for the major donor (i.e., five cells from the major donor) together with some that comprised mini-mixtures consisting of one cell from the minor donor and four cells from the major donor. The latter mini-mixtures with a weight ratio of 1:4 rather than the parental 1:50 bulk mixture would be then be analyzed by the previously validated PG methods for single or few cells [8]. Semi-quantitatively, the modified subsampling strategy expectations for minor donors can be approximated as a binomial probability B(5, 0.02) for a 1:50 mixture where n, the number of trials = the number of cells comprising a single subsample (i.e., five) and the probability of success in finding the minor donor in each single subsample trial is *p* = 0.02. The binomial probability is 0.092, meaning that if one performed 75 subsamplings of 5 cells, then approximately 6–7 of them would result in mini mixtures containing the minor donor. A similar 75 subsamplings for a 1:100 mixture (B(5, 0.01)) would result in an expectation of approximately 3–4 of the 100 subsamplings to be comprised of minor donor containing mini-mixtures.

### 3.2. Recovery of Probative DNA Profile Information from the Minor Donor

**Figure 2:**
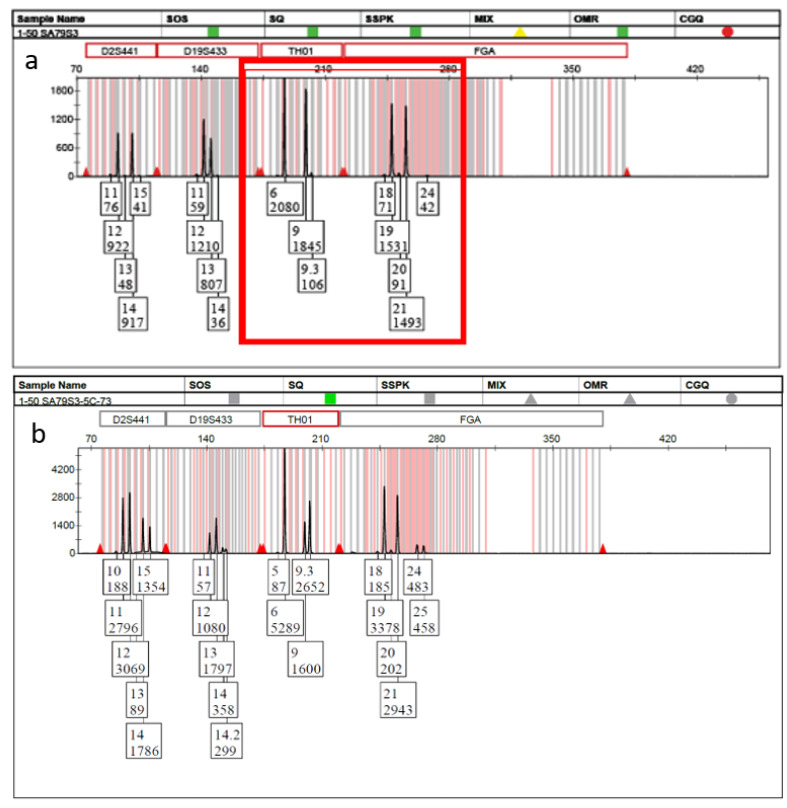
Undetectable/unresolvable minor donor analysis. (**a**) Standard “bulk” approach in which the only indication of a possible minor contributor is the 9.3 and 24 alleles (red box) and (**b**) 5-cell mini-mixture obtained by DSCS indicating the clear presence of a mixture.

Based upon the above subsampling considerations, seventy-five five-cell subsamples were collected from the original 1:50 mixture. In accordance with the binomial sampling expectations, 6 of the 75 collected subsamples consisted of mini-mixtures, which could then be analyzed by the previously validated PG methods for single or few cells [8]. Probative DNA profiles were obtained for the known minor donor within each of the mini-mixture-containing subsamples. Figure 2a shows a single dye channel from part of the electropherogram of the original “bulk” sample. Though the entire DNA profile is not shown, the barely visible THO1 9.3 and FGA 24 alleles (red box) were the only indication of the presence of a minor donor within the entire profile. However, these allelic signals were so low that they could easily be mistaken for noise. Figure 2b shows the same dye channel from one of the five-cell mini-mixture subsamples collected from the same bulk sample and confirms the presence of the second donor.

**Figure 3:**
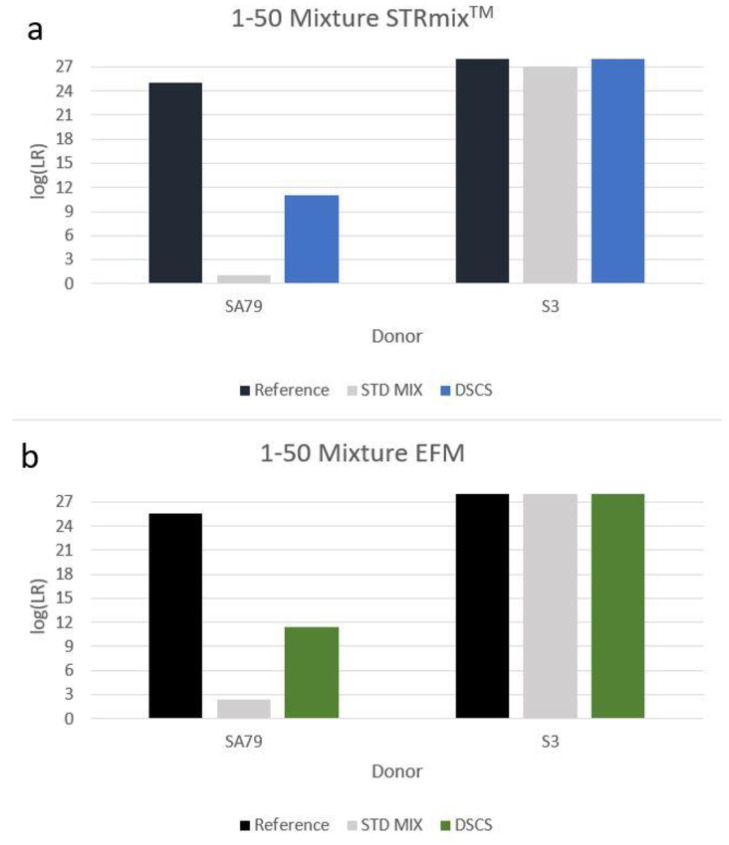
Increased minor donor contributor log(LR) recovery in a 1:50 2-person mixture by DSCS compared to standard PG mixture analysis. STRmix^TM^ (**a**) and EuroForMix (**b**).

PG analysis of the standard bulk 1:50 mixture returned uninformative LRs (according to the internationally recognized verbal qualifiers [18,19]) for the inclusion of the known minor donor (i.e., log(LR)s of ~1–2). However, when PG replicate analysis was conducted on the mini-mixtures obtained, highly probative minor donor log(LR)s (~11) were achieved (Figure 3). Although replicate analysis typically improves the log(LR)s obtained with low template single cell and two cell mini-mixture samples [6], replicate analysis of the 1:4 mini-mixture five-cell subsamples in the 1:50 mixture did not result in a large gain of LR information for the minor donor compared to that obtained for each of the individual five-cell subsamplings. The reasons for this finding are unknown, but we speculate that this is possibly due to the individual subsample log(LR)s in this experiment all being close to the maximum amount of recoverable DNA profile information achievable with this particular minor donor and this particular mixture with the particular subsampling strategy. The minor donor’s DNA comprised one-fifth of the total cell content in a very low template DNA sample (~ 30–33 pg total DNA from five cells) and, as stated, our previous experience with mini-mixture PG replicate analysis was with low template single cell and two cell mini-mixture samples [6]. More studies need to be carried out on quintuple cell subsampling PG replicate analysis with minor donors to determine the extent to which replicate analysis is of use in improving the magnitude of the recovered inclusionary LRs.

## 4. Conclusions

The DSCS approach was applied to mixtures in which a minor donor was virtually undetectable by standard approaches resulting in a probative gain of DNA profile information. Collecting multiple five-cell subsamples permitted the genetic interrogation of a large number of cells (75 × 5 = 375 cells) in an attempt to detect the presence of a minor donor. This subsampling procedure deliberately created mini-mixtures containing cells from the minor donor and resulted, after PG replicate analysis, in highly probative inclusionary minor donor log(LR)s (~11). Although as a proof-of-concept study we took 75 × 5-cell subsamplings, laboratories could choose different subsampling schemes. For example, one could take 35–40 subsamples and evaluate the results to see whether a minor donor was detected (as might be expected if there was a 1:50 barely detectable minor donor mixture) then decide whether it would be necessary or useful to perform another subsampling. In our experiment, we detected the minor donor on the twelfth collected subsample (again in accordance with binomial sampling expectations), therefore 40 × 5-cell subsamples may be sufficient to obtain three replicates of the minor donor.

The approach described could prove useful to not only improve the quality of results obtained from mixtures with an undetectable minor donor when both donors are male or when the minor donor is female, but also could provide additional inclusionary support for true donors in those mixtures that currently need to rely on Y-STR analysis for the detection of the person of interest. Although this proof-of-concept experiment was applied to a binary mixture, similar subsampling schemes to interrogate more complex mixtures with >2 donors (to recover probative DNA profile information from undetectable/unresolvable minor donors) can be envisioned.

## Abbreviations

DSCS	direct single cell subsampling
LR	likelihood ratio
PG	probabilistic genotyping
POI	person of interest
